# Risk perceptions, knowledge and protection practices related to COVID-19 in Dessie City Administration, Northeast Ethiopia: a community-based study

**DOI:** 10.3389/fpubh.2025.1505621

**Published:** 2025-05-09

**Authors:** Husien Nurahmed Toleha, Ewunetie Mekashaw Bayked, Birhanu Demeke Workneh, Teferi Gedif Fenta

**Affiliations:** ^1^Department of Pharmacy, College of Medicine and Health Sciences, Wollo University, Dessie, Ethiopia; ^2^Department of Pharmaceutics and Social Pharmacy, School of Pharmacy, Addis Ababa University, Addis Ababa, Ethiopia

**Keywords:** COVID-19, knowledge, risk perception, protection practice, Dessie, Ethiopia

## Abstract

**Background:**

COVID-19 is a global concern due to its high transmission and mortality rates. Despite governments’ efforts worldwide to control its spread, many people were hesitant to adopt preventive measures. The effectiveness of these measures largely depends on public willingness, which is influenced by their knowledge and perception of risk. Therefore, this study aimed to examine the knowledge, risk perceptions, protection practices, and related factors concerning COVID-19 in the Dessie City Administration, Northeast Ethiopia.

**Methodology:**

This study employed a cross-sectional design. We selected seven hundred ninety participants using a systematic sampling technique. Data was collected face-to-face using an interviewer-administered questionnaire. Descriptive statistics, including frequency, percentage, mean, and standard deviation, were used to summarise the sample characteristics. Multivariable logistic regression analysis was conducted using SPSS (version 23), and the results were presented in the form of text, tables, and graphs.

**Results:**

Of the study participants, 498 (63%) had good knowledge of the pandemic, while 457 (58%) had a low-risk perception. Only 305 (39%) demonstrated good protection practices. The most trusted sources of information were healthcare personnel (686 participants, 86.8%), followed by the Ministry of Health websites (654 participants, 82.8%). Monthly income (>10,000 ETB), knowledge, and risk perceptions with AORs of 3.05 (CI: 1.51–6.14), 4.45 (CI: 2.81–7.04), 2.06 (CI: 1.38–3.08) were significantly associated with protection practices against the COVID-19 pandemic.

**Conclusion:**

More than two-thirds of the participants demonstrated good knowledge about COVID-19. However, over half perceived themselves to be at low risk and engaged in poor preventive practices. Control efforts will be challenging, especially among younger and less educated groups who consider themselves at low risk, requiring focused attention. Understanding people’s risk perceptions and beliefs about the effectiveness of COVID-19 prevention measures is essential for improving protective behaviours. Health education and active community engagement are key strategies in combating the spread of the virus.

## 1 Introduction

Coronavirus disease 2019 (COVID-19) is a contagious respiratory disease in humans that causes pneumonia-like infection (1). So far, seven human coronaviruses (HCoVs) have been identified. These include the highly pathogenic severe acute respiratory syndrome coronavirus (SARS-CoV), Middle East respiratory syndrome coronavirus (MERS-CoV), and the newly identified SARS-CoV-2. They are known to have caused global outbreaks, with SARS-CoV-2 being responsible for the current pandemic (2).

The COVID-19 pandemic emerged in Wuhan, China, in late December 2019 (3). Later, this disease was declared a global public health emergency (4). COVID-19 was declared a pandemic by WHO on March 11, 2020, due to an increase in the number of cases worldwide (5). On March 13, 2020, the first COVID-19 case in Ethiopia was confirmed (6).

Contaminated objects and respiratory droplets were the primary means of transmission for this pandemic (7). Asymptomatic patients serve as carriers of this pandemic (8). Old age and patients with pre-existing chronic illnesses were identified as risk factors for the severe form of the disease and mortality (9). Fever, dry cough, fatigue, respiratory illnesses, diarrhoea, vomiting, and headache were among the clinical manifestations (10).

Non-pharmaceutical interventions (NPIs) are key to controlling the spread of this virus (11), relying on public willingness, which is influenced by knowledge and risk perception (12). However, studies showed inconsistent knowledge about the pandemic. A study in Bangladesh showed that about 45% of participants had poor knowledge (13). A Chinese study showed that droplet infection was mentioned as a mode of transmission by only 66.6% of the participants, while 35.9 and 4.8% of the participants, respectively, mentioned people with ages greater than 65 and smokers as being at high risk of contracting COVID-19 (14).

Because of its high transmission rate, lack of definitive treatment, and high morbidity and mortality rate, this disease is a global concern (15). Controlling its spread was extremely difficult due to the lack of definitive treatments (16). Developed countries are adversely affected, despite their advancement in healthcare systems (17). Ethiopia is an African country with a low-skilled workforce and technological assets (18). Governments all over the world are working hard to keep it under control. However, a large number of people were reluctant to use preventive measures. To be effective, information is crucial to comprehend the relationships between demographic factors, risk perceptions, and prevention practices (19).

Several studies conducted in various countries revealed that the risk perception for this disease was low. A study done in Ghana showed that about 32% of participants had low-risk perceptions (20). A Myanmar study showed that approximately 22% of the participants wrongly perceived spontaneous recovery (14). According to a study conducted in Iraq, only 26.9, 29.7, and 41.7% of respondents, respectively, were concerned about contracting infection, serious illness, or death from this pandemic (21).

The number of COVID-19 cases and deaths in Ethiopia has increased over time. The Ethiopian Ministry of Health reported 102,720 cases as of December 14, 2020 (22). Ethiopia adapts basic intervention measures to reduce the spread of this virus (23). However, people’s adherence to preventive measures is critical, which is influenced primarily by their knowledge and risk perception (3).

Protection practices rely on the perceived risk of contracting a disease and efficacy beliefs for preventive modalities (24). Health models advocate risk perception as a motivator for change in behaviour (25). One of them is the extended parallel process model (EPPM), which advocates protection practice as a result of threat assessment and coping evaluation based on the concept of protection motivation theory (PMT). Threat assessment entails assessing the risk of contracting a disease (perceived vulnerability or susceptibility) and determining the severity of the disease (perceived severity) (26).

Several studies found that protection practices against this disease were not satisfactory. For instance, studies done in Myanmar (14) and Bangladesh (27) revealed that nearly 45.2 and 24.8% of participants, respectively, had poor hand hygiene practices. Studies in the Philippines (28) and Malaysia (29) showed that around 37 and 49% of participants were not avoiding crowded areas, respectively. A Bangladesh (27) study showed that around 30% of participants failed the regular mask-wearing practice. Studies done in Myanmar (14), Bangladesh (27), and Thailand (30) showed that 78, 50, and 83% of respondents had poor protection practices, respectively.

Males, younger age groups, and less educated individuals were less likely to implement preventive measures (31). The clarity of communication influences protection behaviour (32). Risk perception and beliefs about the efficacy of the preventive methods influenced the community’s engagement in realising precautionary measures (33). People with good knowledge and a high-risk perception of the pandemic respond positively to preventive methods (34, 35).

As a result, the purpose of this study was to assess knowledge, risk perceptions, and protection practices in Dessie City, Northeast Ethiopia, as well as investigate factors associated with protection practices.

## 2 Methods

### 2.1 Study area and period

The study was conducted among the population of Dessie City, North-East Ethiopia, between the end of July and mid-December 2020. Dessie is the main trading center in the Northeastern part of Ethiopia and is a part of the Wollo culture, thus having a style of close cultural relationship. The city is divided into 26 kebeles (the smallest administrative unit) for administrative purposes. The city has a number of publicly and privately owned health institutions. The city has one referral hospital that serves more than eight million people from all over the city and provinces. The city also serves as a COVID-19 treatment and quarantine center.

### 2.2 Population and study design

This community-based cross-sectional survey was done among the selected households in the study area. Adult members (>18 years) were selected by lottery from the participating households for an interview. Those with hearing and speaking problems and the inability to comprehend due to illness or age were excluded.

### 2.3 Variables

The outcome variable was protection practice. Its score was determined using a five-point Likert scale. On the scale, one point stands for never, two points for seldom, three points for occasionally, four points for frequently, and five points for always.

Sociodemographic, access to trusted health information, a member with chronic illness (such as diabetes, hypertension, and cancer), knowledge, belief in the efficacy of prevention, and risk perception variables were used as control covariates in the regression models.

Socio-demographic characteristics included sex, age, marital status, religion, education, occupation, family size, and economic status.

Knowledge was measured by 27 items. Furthermore, participants were asked about various sources of knowledge-based information, including social media platforms such as Facebook and others, official websites (WHO websites, MOH updates), official statements on radio and television, family members, and others. Their trust in health information obtained from such sources was also assessed.

Risk perception was measured using 12 items developed according to the framework of the EPPM and WHO COVID-19 survey tool and guidance (26, 36). Perceived efficacy to the desired protection measures addressed their belief in response to efficacy and self-efficacy for the novel COVID-19 (14, 26). The risk perception and belief in efficacy for preventive methods were assessed using a five-point Likert scale. On the scale, one point stands for strongly disagree, two points for disagree, three points for neutral, four points for agree, and five points for strongly agree.

### 2.4 Sample size determination and sampling procedures

The number of households was determined using the single population proportion formula. The sample size was calculated using Epi Info with a 5% margin of error, resulting in 376 participants. However, the sampling procedure involved two stages, and the design effect was considered in addition to a 5% contingency for non-response. The final sample size was 790 households.

**Figure 1:**
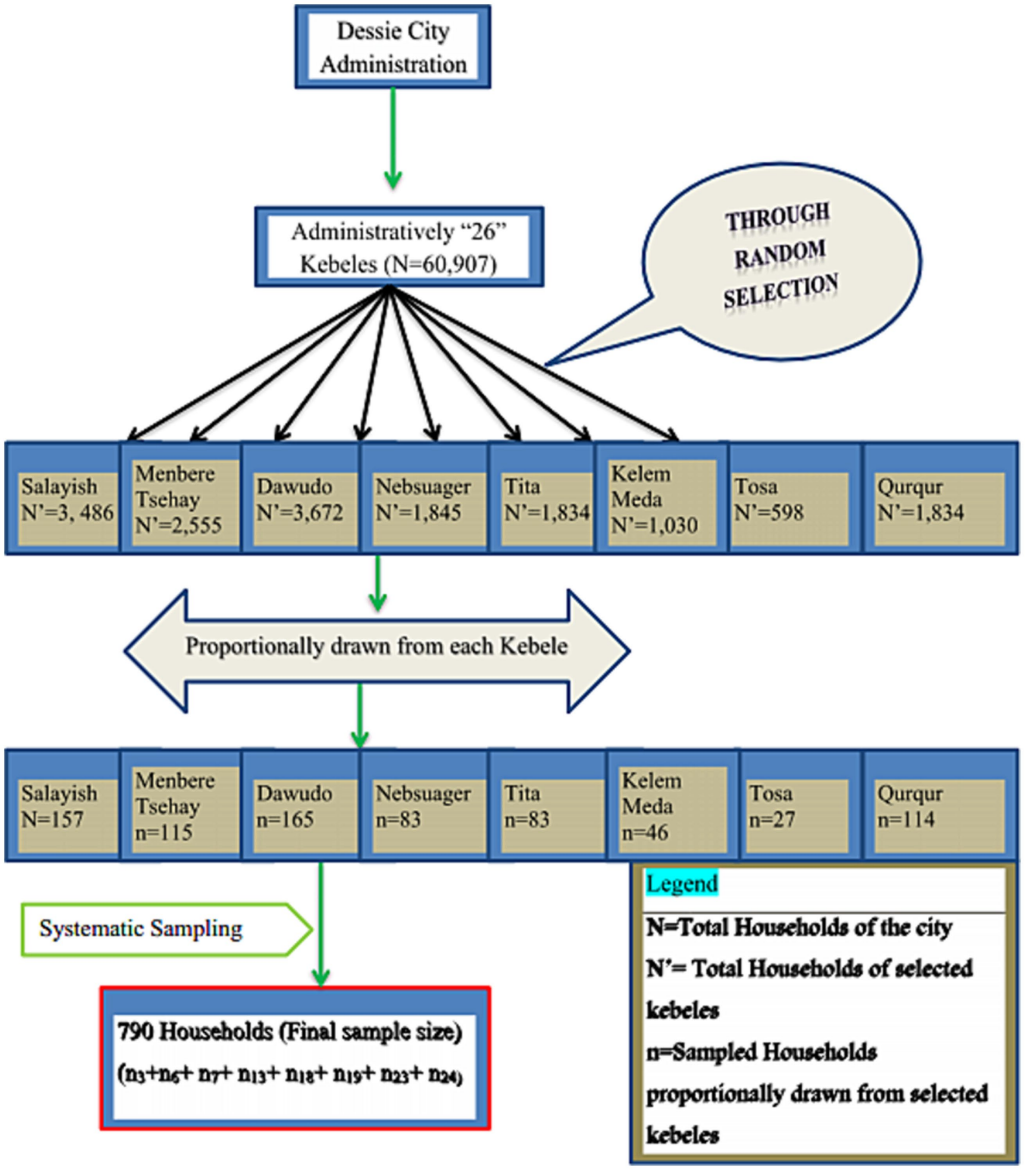
Sampling procedures for assessing risk perceptions, knowledge, and protection practices related to COVID-19, Dessie, Ethiopia, 2020.

In the sampling procedures, first, from the total of 26 “*kebeles*,” eight “*kebeles*” were randomly selected as the first sampling unit using the lottery method. Then the sampled households were proportionally divided into the selected “*kebeles”* according to their number of households. The household list was obtained from “*kebele”* health post extension workers and used as a sampling frame. The first household was selected randomly from the central location of the “*kebeles*.” Then household members from each “*kebele”* were selected through a systematic sampling (Figure 1).

### 2.5 Data collection tools and procedures

A structured interviewer-administered questionnaire was employed for the data collection. The questionnaire was adapted from a previous survey conducted in different settings (12, 14, 21, 37). It was prepared in English and translated to Amharic and back to English by a PhD English language expert for validity. It contained the following parts: (1) socio-demographic characteristics, (2) COVID-19 knowledge items, (3) health information knowledge items, (4) risk perception items, (5) belief in efficacy for the preventive methods, and (6) protection practice items.

The questionnaire contained 27 knowledge items (Table 1). These questions were answered on a yes/no basis, with an additional “I do not know” option.

**Table 1:** COVID-19 knowledge items questionnaire, Dessie, Ethiopia, 2020.

COVID-19 knowledge items	Response options
K1. Do you think COVID-19 exists?	Yes, no, I do not know
K2. Is COVID-19 a viral infection?	Yes, no, I do not know
K3. Can COVID-19 be transmitted from person to person?	Yes, no, I do not know
K4. Could an asymptomatic person transmit the disease?	Yes, no, I do not know
K5. Do you know that COVID-19 has a vaccine?	Yes, no, I do not know
K6. Do you know that there is a cure for COVID-19?	Yes, no, I do not know
K7. Is COVID-19 spread through the air via respiratory droplets produced by infected people sneezing or coughing?	Yes, no, I do not know
K8. Does touching or shaking an infected person’s hands result in COVID-19 infection?	Yes, no, I do not know
K9. Is close contact with people increasing the virus’s spread?	Yes, no, I do not know
K10. Does touching an object and then touching your mouth, nose, or eyes with an unwashed hand result in COVID-19 virus infection?	Yes, no, I do not know
K11. Does using a public toilet result in COVID-19 virus infection?	Yes, no, I do not know
K12. Do people infected with COVID-19 get a fever?	Yes, no, I do not know
K13. Does COVID-19 infection cause dry cough?	Yes, no, I do not know
K14. Do COVID-19 infected people have trouble breathing?	Yes, no, I do not know
K15. Does COVID-19 infection cause headaches?	Yes, no, I do not know
K16. Is it possible to prevent the spread of the virus by washing hands with soap and water for at least 20 s or using an alcohol-based hand sanitizer (60%)?	Yes, no, I do not know
K17. Is contact isolation and quarantine of people infected with the COVID-19 virus effective in reducing virus spread?	Yes, no, I do not know
K18. Does limiting handshakes reduce the risk of infection?	Yes, no, I do not know
K19. Is it possible to prevent the virus from spreading by not touching the eyes, nose, or mouth with unwashed hands?	Yes, no, I do not know
K20. Does avoiding going to crowded places such as public transportation, religious places, hospitals, and workplaces prevent the spread of the virus?	Yes, no, I do not know
K21. Do you know that you should keep a safe distance of at least two meters when sitting with other people to protect yourself from the virus?	Yes, no, I do not know
K22. Do you know that staying at home can reduce your chances of contracting an infection?	Yes, no, I do not know
K23. Do you know that wearing facemasks when leaving the house can help prevent the virus from spreading?	Yes, no, I do not know
K24. Is the older adult (those over the age of 65) more vulnerable to coronavirus disease?	Yes, no, I do not know
K25. Are smokers more vulnerable to the coronavirus disease?	Yes, no, I do not know
K26. Are people with chronic diseases (like hypertension, diabetes, and cancer) more susceptible to coronavirus infection?	Yes, no, I do not know
K27. Are people in densely populated areas more susceptible to COVID-19?	Yes, no, I do not know

This study used six items with five levels of agreement to assess belief in the efficacy of COVID-19 prevention methods. The questionnaire included 12 risk perception items: six for perceived susceptibility or risk of infection (R1–R6) and six for perceived severity (R7–R12) of COVID-19. Negative items were scored in reverse, and the sum of all items was calculated to get the perceived risk score. The questionnaires for risk perceptions, belief in the efficacy of preventive methods, and COVID-19 protection practices are shown in Table 2.

**Table 2:** Questionnaire on COVID-19 risk perceptions, belief in the efficacy of preventive methods, and protection practices, Dessie, Ethiopia, 2020.

Risk perception items	Response options
R1: There is a lower risk of infection spreading to family members from the sick person®	SD, D, N, A, SA
R2: Healthy people have no chance of contracting an infection®	SD, D, N, A, SA
R3: Young people have a low risk of contracting an infection®	SD, D, N, A, SA
R4: People who work with unidentified strangers are vulnerable to infection.	SD, D, N, A, SA
R5: Crowded areas are at risk for disease transmission.	SD, D, N, A, SA
R6: A healthy lifestyle reduces the likelihood of infection.	SD, D, N, A, SA
R7: The COVID-19 virus will cause severe medical problems.	SD, D, N, A, SA
R8: I would not be able to survive if I became infected with COVID-19.	SD, D, N, A, SA
R9: I could have COVID-19 without showing any signs or symptoms®	SD, D, N, A, SA
R10: If I become infected with COVID-19, I will recover on my own®	SD, D, N, A, SA
R11: If I were infected with COVID-19, I could be treated®	SD, D, N, A, SA
R12: I'm worried about the recent COVID-19 outbreak.	SD, D, N, A, SA
Belief in the efficacy of preventive methods	Response options
E1: I can get trustworthy information about the effectiveness of COVID-19 control methods.	SD, D, N, A, SA
E2: A healthy diet effectively prevents COVID-19.	SD, D, N, A, SA
E3: Hand washing or the use of hand sanitizer effectively prevents COVID-19.	SD, D, N, A, SA
E4: I easily implemented self-care measures to prevent COVID-19.	SD, D, N, A, SA
E5: Avoiding crowded areas is an effective way to prevent COVID-19.	SD, D, N, A, SA
E6: When entering a crowded area, wearing a face mask effectively prevents COVID-19.	SD, D, N, A, SA
Participants' frequency of implementing protective measures	Response options
P1: How often do you avoid large crowds?	Nev,Sel,Occ,Freq,Al
P2: How frequently do you avoid touching your face, mouth, nose, and eyes?	Nev,Sel,Occ,Freq,Al
P3: How frequently do you limit contact (such as handshakes)?	Nev,Sel,Occ,Freq,Al
P4: How frequently do you avoid unnecessary travel or trips?	Nev,Sel,Occ,Freq,Al
P5: How frequently do you avoid close contact with sick or infectious people?	Nev,Sel,Occ,Freq,Al
P6: How frequently do you wash your hands for at least 20 seconds or use hand sanitizer with 60% alcohol?	Nev,Sel,Occ,Freq,Al
P7: When you sneeze or cough, how often do you use a tissue?	Nev,Sel,Occ,Freq,Al
P8: How frequently do you wear face masks in public?	Nev,Sel,Occ,Freq,Al
P9: How often do you throw away used masks or tissues in the trash?	Nev,Sel,Occ,Freq,Al
P10: When you're sick, how often do you stay at home?	Nev,Sel,Occ,Freq,Al
P11: How often do you practice "physical distancing" by standing 2 meters away from others?	Nev,Sel,Occ,Freq,Al

SD = Strongly Disagree; D = Disagree; N = Neutral; A = Agree; SA = Strongly Agree Nev=Never; Sel=Seldom; Occ=Occasionally; Freq=Frequently; Al=Always.

Data was collected between the end of July and the middle of December 2020. Two trained data collectors collected the data using a structured one-on-one interview technique. During the data collection, respondents were educated on adherence to standard precautions and PPEs such as antiseptic fluids, face masks, and latex gloves. All necessary precautions were taken to prevent disease transmissibility during data collection.

### 2.6 Quality assurance

The study was pre-tested on 40 respondents before the actual data collection. The survey tool was amended for consistency. Trained data collectors with a diploma in pharmacy collected the data. The investigator provided comprehensive one-day training about the data collection instruments, approaches required for interaction, and securing respondents’ consent.

Cronbach’s alpha was used to assess the reliability of the interviewer-administered questionnaire, and values of 73.6, 71.5, and 81.9%, respectively, were obtained for the knowledge, risk perception, and protective practice item questionnaires. This indicates that the internal consistency is acceptable (38).

The logistic regression test assumptions were verified and met the criteria (a binary dependent variable, independent observations, multicollinearity, outliers, and a large sample size). The Box-Tidwell test was used to check the linearity assumptions. The model was fitted for the assumptions.

### 2.7 Statistical analysis and presentation

Descriptive analysis was used to generate sample characteristics. The data was summarised using frequency, percentage, mean, and standard deviation. First, the bivariable binary logistic regression analysis using SPSS (version 23) was used to determine the relationship between each independent variable and the outcome variable in order to identify factors associated with protection practice. The variables with a *p*-value < 0.2 in the bivariate analysis were entered into the multiple logistic regression model to identify the independent factors of the outcome variable. The associations between independent variables and protection practices were quantified using crude odds ratios (CORs) and adjusted odds ratios (AORs) with 95% confidence intervals (CIs). In the final model, variables with a *p*-value < 0.05 were significant. The findings of the study were then presented in the form of text, tables, and graphs.

### 2.8 Operational definitions

The mean value is used as a cut-off value when defining knowledge, risk perceptions, belief in the efficacy of preventive methods, and protection practices. The mean, which is the average value from each observation in the dataset, identifies the center of a dataset. As a result of these considerations, the mean was chosen as the cutoff value for the study. In addition, previous research has used the mean value as a cut-off point when categorising such variables (12, 39, 40).

#### 2.8.1 Knowledge scores

When the scoring was greater than the mean score (18.9) of items, it indicated good knowledge, while scores below the mean indicated poor knowledge of the COVID-19 pandemic.

COVID-19 risk perception *was* measured using items for threat appraisal (susceptibility and severity). *Susceptibility refers to* respondents’ belief that they are infected with the disease. *Severity* refers to respondents’ perceptions of the seriousness of the disease if infected with COVID-19. If a risk perception score exceeded the mean score (36.03) of the items, it was considered high; when it was less, it was considered low.

#### 2.8.2 Belief in efficacy for the preventive methods

The belief in efficacy of preventive methods was measured using items for efficacy appraisal (response efficacy and self-efficacy) of preventive modalities. *Perceived efficacy* refers to participants’ belief in the efficacy of preventive modalities and their ability to act.

#### 2.8.3 Protection practice scores

A score greater than the mean score (29.06) of items indicated good practice, while a lower score indicated poor practice.

## 3 Results

### 3.1 Socio-demographic characteristics

Seven hundred ninety participants enrolled with a 100% response rate. Table 3 depicts the socio-demographic characteristics of the study’s participants. More than half, 433 (54.8%), of the study participants were female, and 464 (58.7%) were married. In terms of religion, Muslims made up more than half, 419 (53%), of the study participants, with Orthodox coming in second. The average age of the participants was 40.8 years, with ages ranging from 21 to 68 years. The most common age range, 338 (42.8%), was 31 to 40 years old. In terms of education, 461 (58.4%) of the study participants had a secondary education or less, while 191 (24.2%) had a bachelor’s degree or above. A large proportion, 268 (33.9%), of the participants’ monthly income ranged from 2,001 to 3,000 ETB. Regarding the medical conditions, 137 (17.3%) of the study participants had at least one or more chronic diseases such as diabetes, hypertension, or lung disease (Table 3).

**Table 3:** Socio-demographic characteristics of the study population, Dessie City, Ethiopia, 2020.

Variables	Description	Frequency	%
Gender	Male	357	45.2
	Female	433	54.8
Age (years)	<31	136	17.2
	31–40	338	42.8
	41–50	146	18.5
	51–60	111	14.1
	>60	59	7.5
Religion	Islam	419	53
	Orthodox	322	40.8
	Protestant	49	6.2
Marital Status	Single	223	28.2
	Widowed	73	9.2
	Divorced	30	3.8
	Married	464	58.7
Education	No formal education	152	19.2
	Primary school	145	18.4
	Secondary school	164	20.8
	Diploma	138	17.5
	Bachelor-degree or above	191	24.2
Occupation	Unemployed	112	14.2
	Student	43	5.4
	Private-employee	299	37.8
	Housewife	68	8.6
	Merchant	103	13
	Governmental employee	165	20.9
Monthly income (ETB)	<2,000	148	18.7
	2,001–3,000	268	33.9
	3,001–5,000	177	22.4
	5,001–10,000	104	13.2
	>10,000	93	11.8
Chronic illness	No	653	82.7
	Yes	137	17.3

ETB, Ethiopian Birr.

### 3.2 Knowledge about COVID-19

COVID-19 was thought to exist in Ethiopia by 713 (90.3%) of participants, and the virus was mentioned as the cause of this disease by 691 (87.5%). Around nine to ten, or 710 (89.9%), of the respondents stated that it was transmitted from person to person. A large proportion of study participants, 540 (68%) and 615 (78%), stated that there was no vaccine or definitive treatment for COVID-19, respectively.

Seven hundred thirty-three (92.8%) and 655 (82.9%) of the study participants agreed that respiratory droplets from infected individuals during coughing, sneezing, or expiration, as well as close contact with the people, would result in the virus spreading, respectively. Shaking an infected person’s hand and touching a virus-infected object were also mentioned as ways of COVID-19 transmission by 624 (79%) and 596 (75.4%) of the participants, respectively.

Fever, dry cough, difficulty breathing, and headache were declared by study participants to be the most likely symptoms of infected individuals by 760 (96.2%), 707 (89.5%), 690 (87.3%), and 655 (82.9%), respectively. Six hundred seventy-seven (85.7%) and 635 (80.4%) participants stated that people in crowded places and those with chronic diseases were at high risk of contracting COVID-19, respectively.

Face masks and frequent hand hygiene were mentioned as important preventive measures by 710 (89.9%) and 699 (88.5%) of participants, respectively. Six hundred thirty-six (80.6%) and 612 (77.5%) participants reported that avoiding crowded places and contact isolation reduced the spread of the virus, respectively (Table 4).

**Table 4:** Knowledge regarding preventive measures for COVID-19 among participants in Dessie, Ethiopia, 2020.

Knowledge of COVID-19 preventive modalities	Frequency	Percentage
Put on face masks	710	89.9
Handwashing with soap and water every 20 s or using an alcohol-based hand sanitizer	699	88.5
Avoid crowded places such as public transportation, religious places, hospitals, and workplaces	637	80.6
Contact isolation and quarantine of infected individuals	612	77.5
Limiting handshakes	610	77.2
Avoid touching your eyes, nose, and mouth with unwashed hands	593	75.1
Staying at home	550	69.6
Maintain at least a two-meter safe distance	531	67.2

The study participants’ average knowledge score was 18.9 (range: 9 to 25). Approximately two-thirds of the participants in the study, 498 (63.0%) (95% CI (59.7–66.4%)), had a good level of knowledge (i.e., greater than the mean score of 18.9).

### 3.3 COVID-19 health information sources

**Figure 2:**
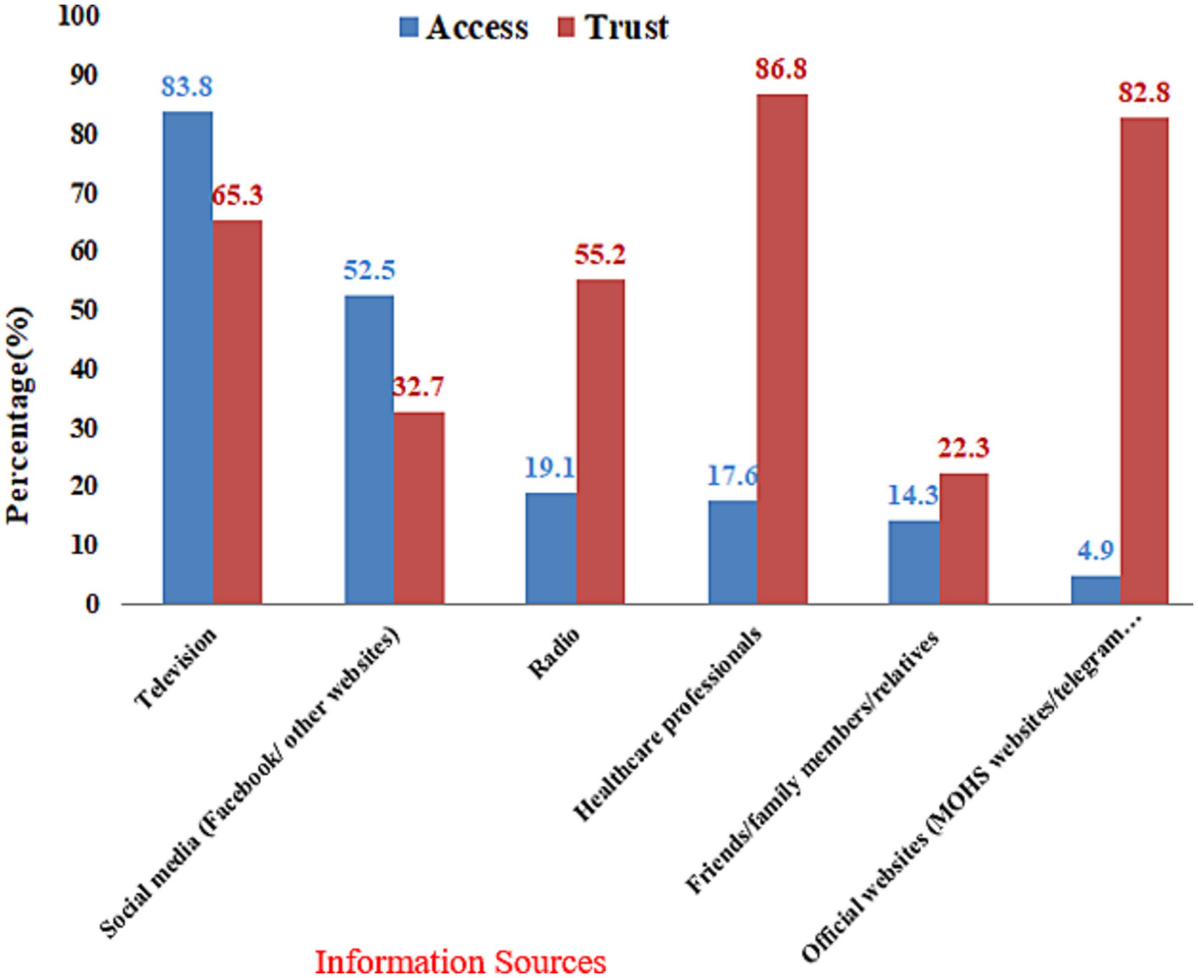
Sources of health information access and trust among the participants, Dessie City, Ethiopia, 2020.

The most common sources of health information about COVID-19 were television (662, 83.8%) and social media (415, 52.5%). Health care personnel were the most trusted sources, 686 (86.8%), followed by information obtained from MOHS websites, 654 (82.8%), and television, 516 (65.3%). Health professionals and MOH officials websites, on the other hand, were the least accessible source of information (Figure 2).

### 3.4 Risk perceptions about COVID-19

**Figure 3:**
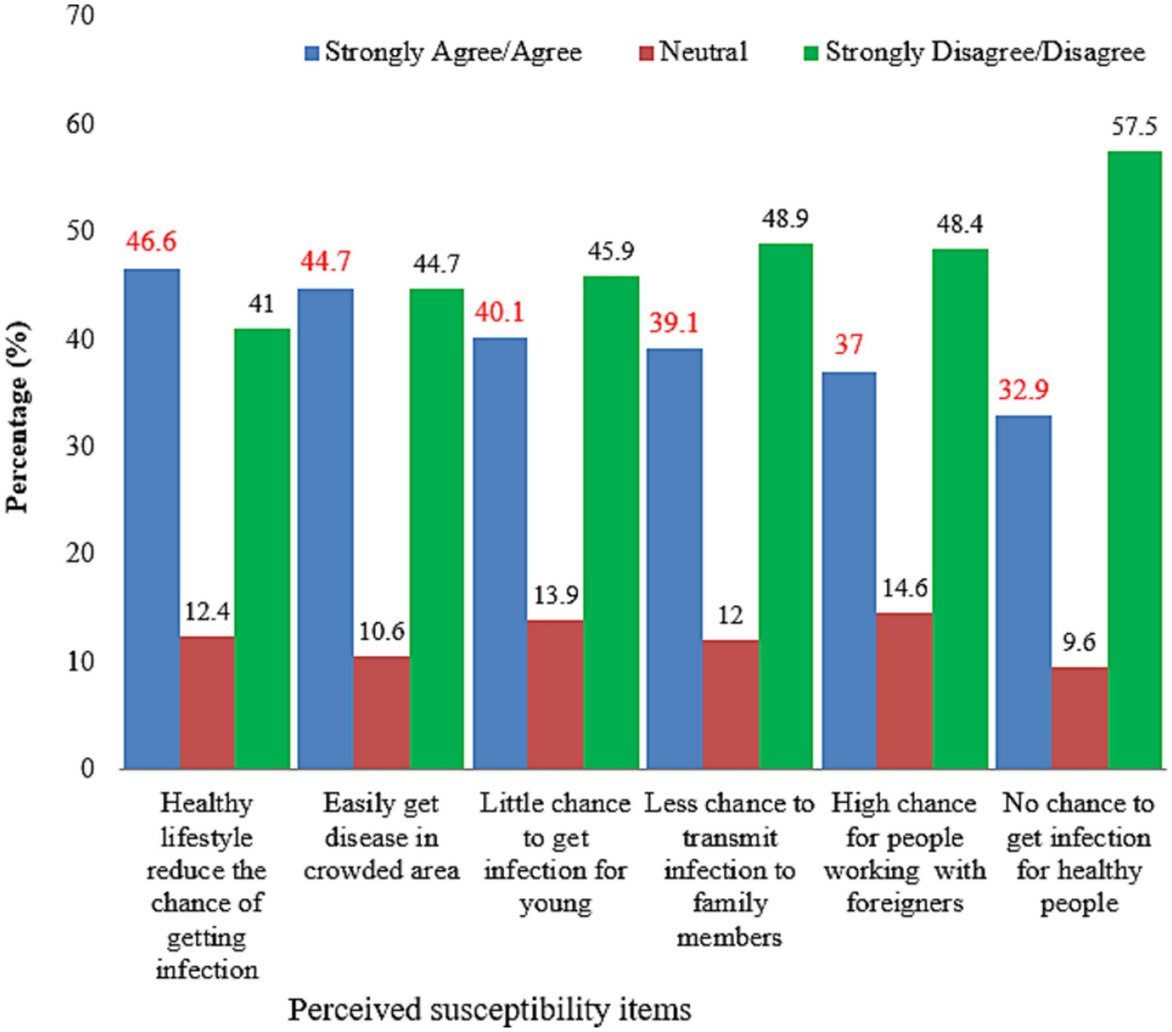
Perceived susceptibility to COVID-19 among participants, Dessie City, Ethiopia, 2020.

The results revealed that 460 (57.8%) of respondents fell into the low-risk perception category, while the remaining fell into the high-risk perception category. Four hundred eight (51.6%) of the study participants perceived they were at low risk of getting an infection. Three hundred seventeen (40%) and 260 (33%) of the respondents incorrectly perceived that there was a low chance of infection among young people and no chance of infection among healthy people, respectively (Figure 3).

**Figure 4:**
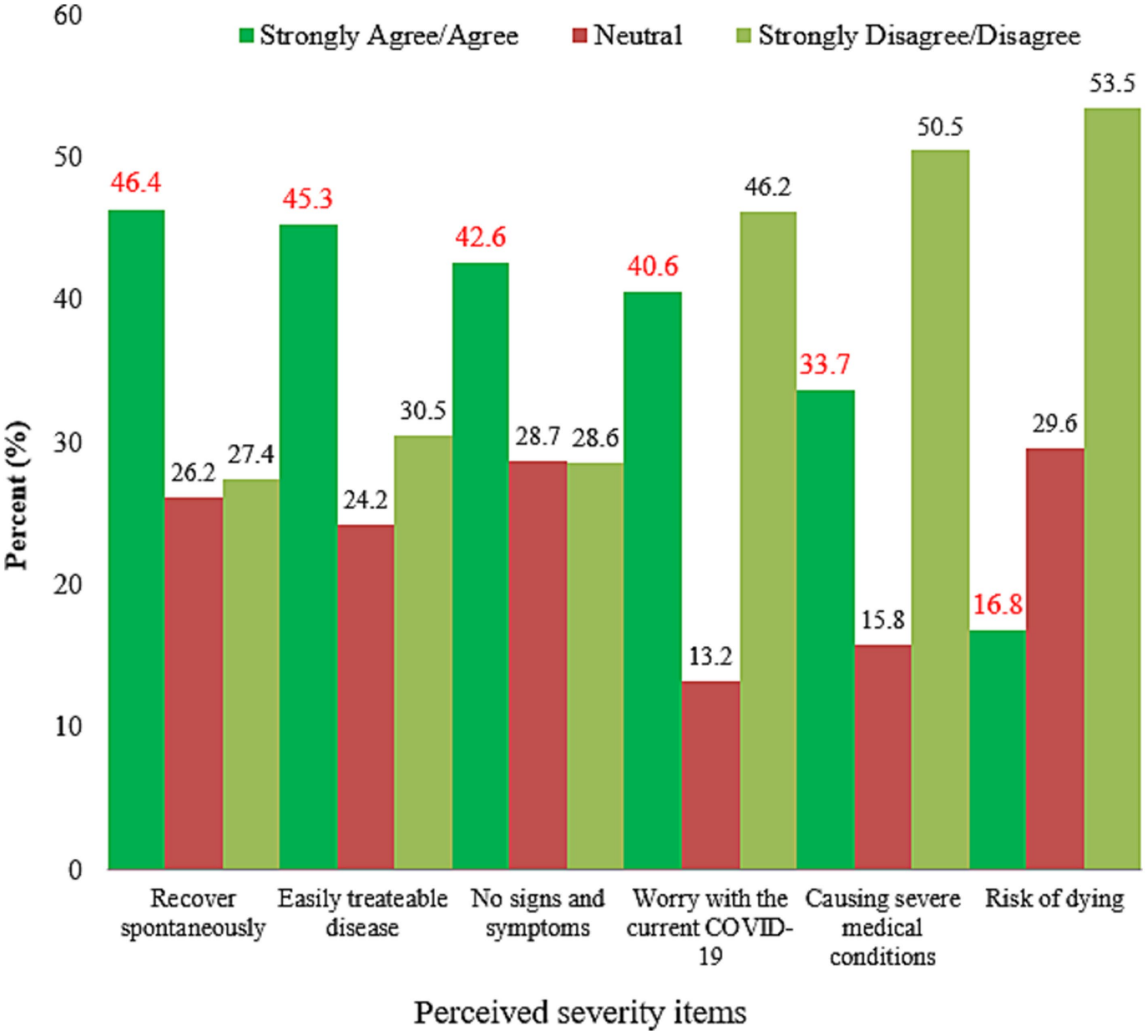
Perceived severity of COVID-19 among participants, Dessie City, Ethiopia, 2020.

Only 266 (33.7%) participants believed COVID-19 would cause severe medical problems, while about 423 (53.5%) thought infected people could survive. Approximately two to five respondents, 337 (42.6%) and 367 (46.4%), believed that this disease was asymptomatic and spontaneous recovery was possible, respectively (Figure 4).

### 3.5 Protection practices and associated factors

#### 3.5.1 Belief in the efficacy of COVID-19 preventive methods

**Figure 5:**
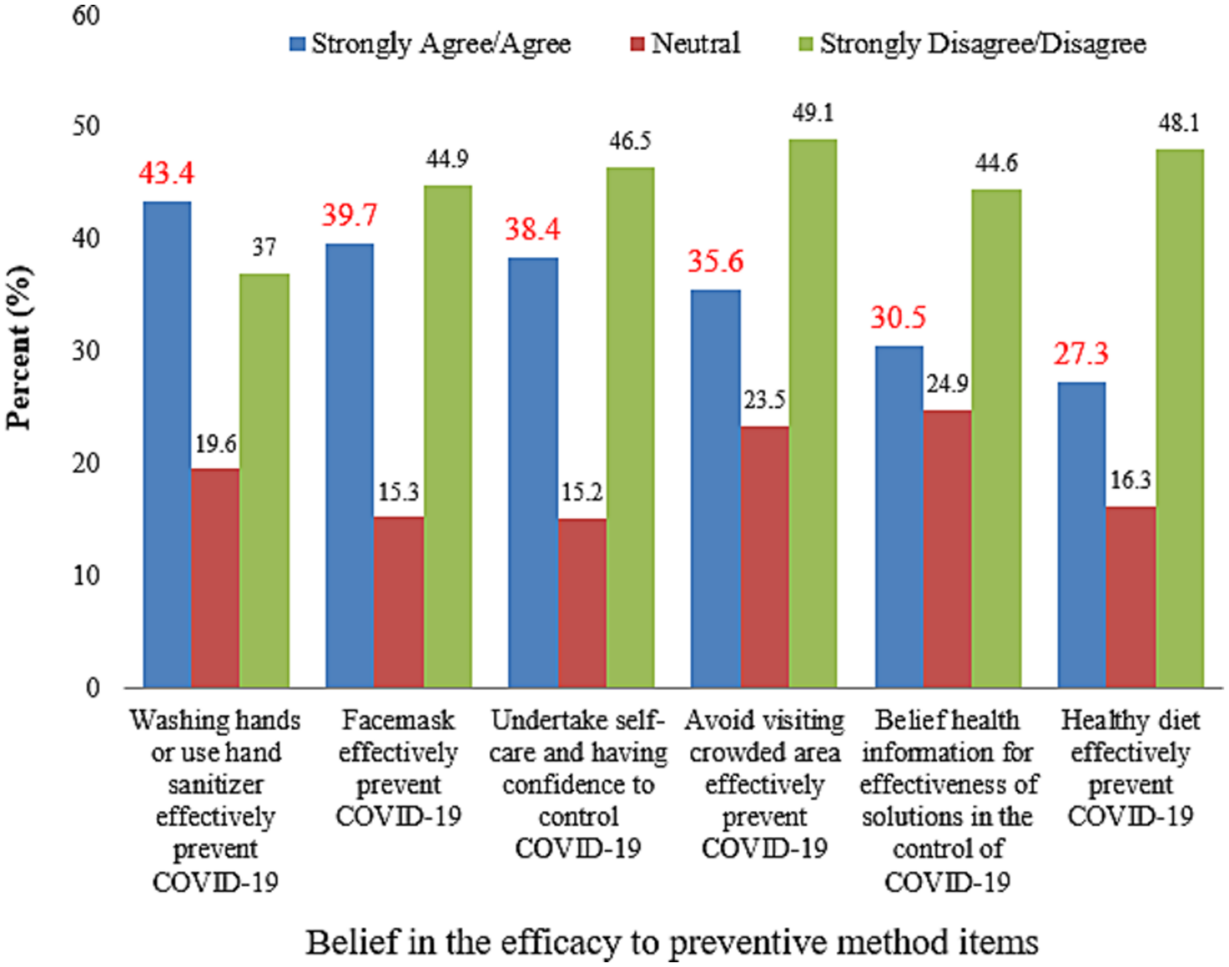
Belief in the efficacy of COVID-19 preventive methods among respondents, Dessie City, Ethiopia, 2020.

Two hundred forty-one (30.5%) of respondents believed they could access reliable health information regarding COVID-19 effective preventive methods. Only 303 (38.4%) of them were motivated to undertake self-care in controlling the spread of COVID-19 using preventive measures. Approximately half of the respondents, 380 (48.1%), were not concerned about their crowded area of visit (Figure 5).

#### 3.5.2 Protection practices

**Figure 6:**
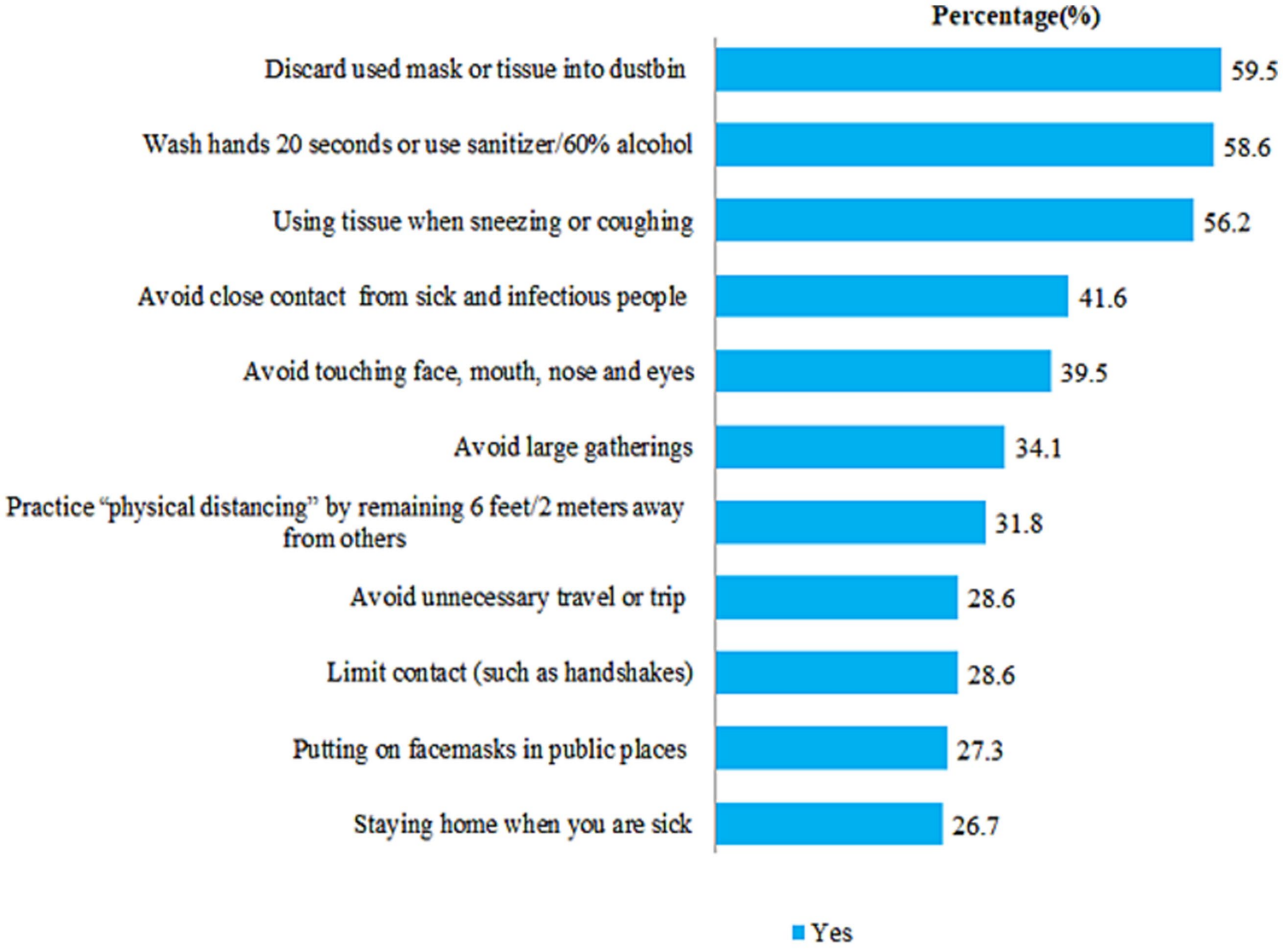
COVID-9 protection practices among participants, Dessie City, Ethiopia, 2020.

The prevalence of good practice was 305 (38.6%), with a 95% confidence interval of 35.0–42.0%. Four hundred sixty-three (58.6%) of participants stated that they wash their hands. The majority of the study participants, 579 (73.3%) and 574 (72.7%), did not stay at home when they were sick and did not wear face masks in public places, respectively (Figure 6).

**Figure 7:**
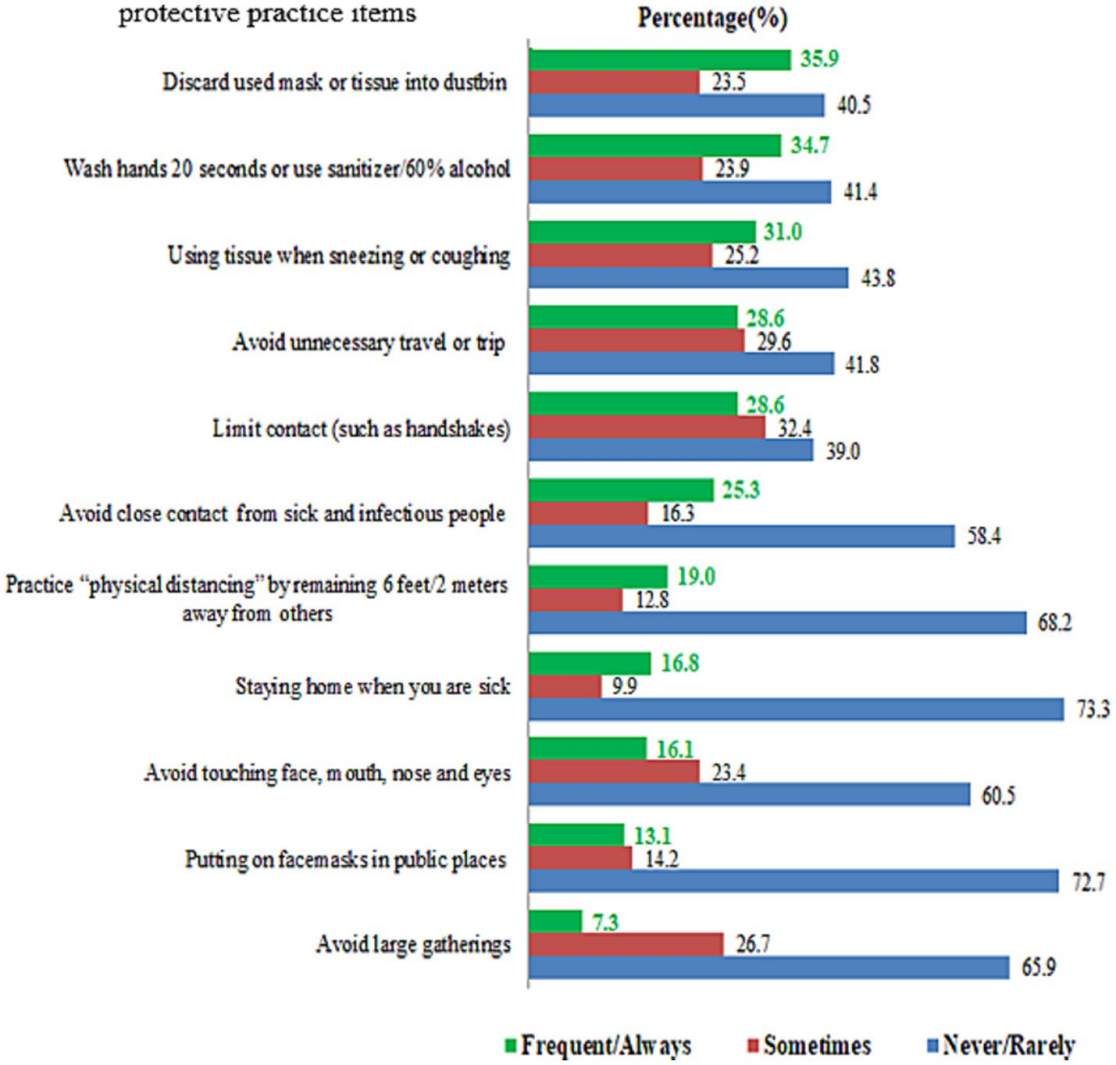
Frequency of protection practices in the community for COVID-19, Dessie City, Ethiopia, 2020.

Only 284 (35.9%) and 274 (34.7%) of the participants frequently discarded used masks into the trash and washed their hands with soap and water, respectively. According to the frequency of use, only 245 (31%) of respondents used tissues when sneezing, and 200 (25.3%) avoided close contact with sick or infectious people (Figure 7).

#### 3.5.3 Factors associated with the protection practices of COVID-19

Socio-demographic variables such as age, educational status, and monthly household income were found to be associated with good protection practices. Knowledge, risk perception, and belief in the efficacy of preventive methods were predictors of good protection practices. Participants with good knowledge levels and high-risk perceptions were more likely to exhibit good protective practices, with AORs of 4.45 (CI: 2.81–7.04) and 2.06 (CI: 1.38–3.08), respectively. Participants with “diploma level qualifications” and “degree or above level qualifications” were 2.04 times (CI: 1.08–3.83) and 2.94 times (CI: 1.64–5.28) more likely to have good protective practices than those with no formal education, respectively. The odds of good practice were 2.59 (AOR = 2.59, CI:1.31–5.15) and 3.05 (AOR = 3.05, CI:1.51–6.14) higher in study participants with household incomes of 5,001–10,000 ETB and ≥10,000 ETB than in participants with a low level of household income (i.e., <2000 ETB), respectively (Table 5).

**Table 5:** Factors associated with the practice of precautionary measures against COVID-19 among participants, Dessie City, Ethiopia, 2020.

Variables	Description	*p*-value	COR (CI)	*p*-value	AOR (CI)
Age (years)	<31		1		1
	51–60	0.000	3.07 (1.82–5.16)		
	>60	0.000	3.52 (1.86–6.66)	0.016	2.31 (1.17–4.59)
Education	No formal education		1		1
	Diploma-level	0.000	3.97 (2.41–6.54)	0.026	2.04 (1.08–3.83)
	Bachelor-level or above	0.000	4.14 (2.59–6.59)	0.000	2.94 (1.64–5.28)
Family Size	<3.5		1		
	>3.5	2.13	0.000 (1.59–2.86)		
Income (ETB)	<2,000		1		1
	5,001–10,000	0.000	2.82 (1.67–4.75)	0.007	2.59 (1.31–5.15)
	≥10,000	0.000	3.63 (2.11–6.25)	0.002	3.05 (1.51–6.14)
Knowledge	Poor		1		1
	Good	0.000	7.66 (5.22–11.24)	0.000	4.45 (2.81–7.04)
Risk perceptions	Low		1		1
	High	0.000	4.35 (3.21–5.91)	0.000	2.06 (1.38–3.08)
Belief in efficacy	Poor		1		1
	High	0.000	7.71 (5.54–10.74)	0.000	5.22 (3.45–7.88)

AOR, Adjusted odds ratio; COR, crude odds ratio; ETB, Ethiopian Birr.

## 4 Discussion

COVID-19 poses a serious economic as well as public health threat. COVID-19 is effectively controlled by increasing public knowledge, perception, and prevention practices.

In line with the previous study (41), nearly nine out of ten participants believed that the virus caused this pandemic (87.5% vs. 96%). The majority of them reported fever, dry cough, difficulty breathing, and headache as symptoms of this pandemic. These findings agreed with those of Fang et al. (42).

The study found that 63% of the participants had good knowledge about the COVID-19 pandemic. This was comparable to findings from Bangladesh (63% vs. 61.4%) (27) but higher than in Myanmar, where the majority (87%) of the participants had poor knowledge (14), and lower than in India (43), Tanzania (44), China (12), and USA (45), where 80.64, 84.4, 90, and 80% of participants, respectively, had good knowledge. The variation in knowledge levels across studies may be due to differences in study population, timing of study, and access to information, highlighting the need for improved health education in the community.

Regarding the source of information, the majority of participants obtained COVID-19-related information from national TV, social media, and radio, with social media being a significant source, similar to studies in Myanmar (14) and Hong Kong (12), approximately half of participants obtained information about COVID-19 from social media. However, this finding differed from the Chinese study, in which the vast majority of participants (98%) obtained information about COVID-19 via social media (46). This shows a high proclivity for people to use social media in recent years. However, trust in the truthfulness of social media information was low. The most reliable information sources were health professionals, MOH official websites, and mass communication tools such as television or radio. These findings were imperative since they may indicate that more efforts should be made to deliver trustworthy information through the most popular and trusted communication channels.

Approximately two out of five participants thought they were at risk of COVID-19. This finding is lower than that of Thailand (75%) (30), Southwest Ethiopia (53.4%) (39), Ghana (68.3%) (20), and Sierra Leone (75%) (47). Furthermore, the study found a higher risk perception than 7.6% in India (48). This could be due to the differences in the study period, study population, and case count. The perceived severity of the disease was lower than in the Hong Kong (49) and Myanmar (14) studies, where almost all participants perceived the disease to be very severe. In this study only 16.8% of the participants thought COVID-19 was a deadly disease, whereas in Thailand, approximately 70% thought it was a severe and dangerous disease (30). Similarly, studies in China found that the majority of people thought COVID-19 was extremely severe (46, 50). In terms of survivorship, if infected, the communities in this study had a higher (53.5%) response rate than Myanmar (22%) (14) and Hong Kong (18%) (12). This explains why the people in this study setting become more hesitant to take preventive measures and require more convincing of the COVID-19 risk. According to a study conducted in Egypt (3), the majority of participants thought the disease was more dangerous for the older adult and those with chronic diseases. This could be because the risk of death from COVID-19 is higher in the older adult and those with medical conditions (51).

Perceived efficacy toward preventive measures such as hand hygiene, wearing face masks, and avoiding crowded areas was lower than in previous studies conducted in Myanmar and Hong Kong (i.e., 49.1% vs. 80 and 90%, respectively) (12, 14). The belief in the efficacy of preventive methods is important information for improving epidemic control. This indicates that more work is required to ratify belief in efficacy.

Handwashing practices were lower in studies conducted in the Philippines (28), Hong Kong (49), and Bangladesh (52) (58.6% vs. 89.9, 95.8, and 98.6%, respectively), but higher than a study conducted in Myanmar (14) (58.6% vs. 44.9%). Avoiding crowded places was lower in this study than in previous studies from Myanmar (14) and Hong Kong (49) (34.1% vs. 57.9 and 88.1%, respectively). Furthermore, the practice of avoiding crowded areas was lower than in a study conducted in the Philippines (28), Bangladesh (13), Tanzania (44), China (12), Nepal (53), and Malaysia (29), where 62.9, 87.97, 77, 96.4, 94.9, and 51.2% of people avoid social gatherings, respectively.

Only 26.7% of participants followed the principles of staying at home. This was much lower than in the Bangladesh study (52), where the majority (93.2%) of respondents followed the stay-at-home principle. Cough etiquette practice was markedly lower than in a Hong Kong study (49) (56.2% vs. 97.1%), but higher than in a Myanmar study (14) (56.2% vs. 47.3%). Only 27.3% of participants put on a face mask when leaving their home. It was much lower than in studies conducted in Bangladesh (13), Malaysia (29), Tanzania (44), China (12), and Nepal (53), where nearly 75, 83.4, 80, 98, and 88.2% of the participants used a face mask. This disparity was attributed to socioeconomic factors, culture, case count, beliefs in the efficacy of preventive measures, and perceptions of COVID-19 risk.

The practice of COVID-19 prevention measures in this study was 38.6%, which was much lower than the study conducted in Iran (54), 71%, and Bangladesh (27), 51.6% of participants had good COVID-19 prevention practices, but higher than the study conducted in Myanmar, 22% (14). The disparities could be explained by differences in information-seeking behaviour, case counts, death rates, misconceptions, and study time.

In line with the study done in Lebanon (41), Iran (55), India (43), Hong Kong (49), and Malaysia (56), education was a strong predictor of protection practices. Behavioural models advocate education is an influential determining factor of healthy behaviour (57). Education leads to better information-gathering habits and efficient use of health inputs (58). This may explain why more educated respondents engaged in precautionary behaviours. This underlines the importance of education in an attempt to raise protection.

In line with the study done in China (12), high income was associated with good practices. Economic status is the primary determinant of actions for maintaining one’s health (57). It has been demonstrated that the low-income households do not use preventive methods (59). In addition, income determines the likelihood of purchasing personal protective equipment, such as a face mask and hand sanitiser. Individuals with low income fail to observe preventive methods; instead, they prefer to continue their daily activities to satisfy their basic needs during the transmission period. This may explain why high-income groups had good preventive practices. Therefore, the government should recognise income discrepancies and provide targeted support to decline the transmission rate across the income spectrum.

Protective practices were predicted by knowledge, aligning with findings from other studies (12, 60). Knowledge enhances awareness, dispels misconceptions, and supports informed decision-making. However, its effectiveness can be shaped by cultural beliefs to the pandemic, socioeconomic conditions, and trust in health systems. Traditional practices, resources limitation, and distrust may hinder the adoption of scientific protective behaviours. In line with other studies (61), risk perception also strongly predicts protective behaviour. People with a low perception of risk should be a prime target for public health education. Additionally, belief in the effectiveness of preventive methods was the strongest predictor of adherence to protective behaviours during the pandemic.

These findings showed that protective behaviour implementation largely depends on community engagement in the prevention pathway. Public health education should focus on enhancing knowledge while addressing key barriers, particularly among individuals with low risk perception and limited belief in the effectiveness of preventive measures, to ensure effective control over the spread of this virus.

## 5 Strengths and limitations of the study

### 5.1 Strengths of the study

In the study setting, although studies had examined knowledge and protective practices for COVID-19, those comprising risk perception in their work were limited. Most previous studies were online application-based surveys; as a strong point, this research was a field-based study. The use of a field survey in the data collection process in this study may have resulted in an adequate representation of the less educated groups. This study solves the issue of representation and the methodological concerns of previous studies.

### 5.2 Limitations of the study

The cross-sectional nature of this study might make it tougher to establish a cause-and-effect relationship. Furthermore, the study is limited to one city administration in Northeast Ethiopia rather than the entire country, which is a limitation.

## 6 Conclusion

The study found that two-thirds of the participants had good knowledge. However, there was a low perception of the risk and poor practices for preventing COVID-19. There is an urgent need to fill the risk perception and knowledge gap to control the expansion of the COVID-19 outbreak.

This study revealed that COVID-19 knowledge, efficacy beliefs, and risk perception significantly predicted precautionary behaviour. Older people, people with a high income, a high level of education, and a large family size readily showed a high propensity toward protection practices. As a result, the young population, as well as lower-income and education groups, would be challenging to the control efforts and will require special attention. Risk communication and community engagement efforts should be considered in fighting against this pandemic.

The results of this study indicate the focus areas that need to be addressed and disclose the need for continued monitoring. The media should make it easier to get reliable health information. Moreover, future research would identify the possible barriers to adopting protective behaviours in fighting the pandemic disease and their solutions.

## Data Availability

The original contributions presented in the study are included in the article/supplementary material, further inquiries can be directed to the corresponding author.
